# Research on multilateral collaboration strategies in agricultural seed quality assurance

**DOI:** 10.1038/s41598-024-61505-5

**Published:** 2024-05-17

**Authors:** Yanmei Wang, Yusheng Chen

**Affiliations:** Management College, Ocean University of China, Qingdao, Shandong China

**Keywords:** Seed quality assurance, Regulatory framework, Impact mechanisms, Evolutionary game, Simulation analysis, Computational biology and bioinformatics, Plant sciences

## Abstract

Seeds, as the initial products in agricultural systems, play a pivotal role in ensuring quality, fundamental to national food security and sustainable agricultural development. This study introduces a concept integrating public governance and evolutionary game theory to construct a quadripartite evolutionary game model involving seed companies, certification agencies, farmers, and governmental departments. It considers the strategic choices of these stakeholders under varying economic motivations and market mechanisms, as well as the influence of external regulation and incentives on game strategies. The existence conditions for evolutionarily stable strategy combinations are determined using the Lyapunov first method, and MATLAB is employed for numerical simulation analysis to validate the game analysis under initial conditions. The simulation results reveal two potential equilibrium points corresponding to different strategic choices among stakeholders. The study finds that producing high-quality seeds and the refusal of certification agencies to engage in rent-seeking are crucial for ensuring seed quality. Additionally, the cost–benefit ratio of seed companies, the speculative cost of certification agencies, and the rights-protection cost of farmers are key determinants in the evolution of seed quality assurance strategies. This research also holds practical significance in enhancing seed quality assurance mechanisms and fostering sustainable development in agriculture.

## Introduction

Agricultural production encompasses numerous facets including land management, fertilizer utilization, and pest and disease control, with seeds being the inception point for these aspects. The regulation of seed quality emerges as a critical segment within the entire agricultural system^1^. High-quality seeds are defined as those that meet or exceed certain quality standards, characterised by a high germination rate, vigorous vitality, appropriate moisture content, high purity, superior genetic traits, and favourable physical properties. From the perspective of seed quality, high-quality seeds significantly enhance both the yield and quality of agricultural produce, in addition to augmenting the crops' resistance against adverse environmental conditions and pests and diseases^2^. A comprehensive optimization of crop growth cycle, disease resistance, and resilience to adverse conditions amplifies the economic and ecological benefits of agricultural system^3^. From the perspective of food security, amidst the backdrop of rapidly escalating global food demand, leveraging high-quality seeds to boost the crop yield per unit area has morphed into an effective measure and a pivotal pathway to addressing food scarcity issues and ensuring national food security^4^. This not only alleviates the pressure on food supply but also elevates the nutritional value and safety of food, catering to the burgeoning consumer demand for healthful food products^5^. From the vantage point of sustainable agriculture, high-quality seeds efficaciously curtail the utilization of agricultural inputs such as fertilizers and pesticides, thereby reducing the pressure of agricultural activities on the environment and natural resources^6^. Consequently, seed quality bears a direct nexus to farmer livelihoods, national food security, and the health and sustainability of agricultural ecosystems. Agricultural seeds industry, as a strategic, foundational core industry, have their quality assurance serving as the linchpin for bolstering agricultural systems^7^. However, incidents of counterfeit and substandard seeds remain recurrent, such as the “over 440 mu reduction in broad bean yield” in Chengjiang, Yunnan in 2022 and the “total crop failure of a hundred mu of chili” in Xiangyang, Henan in 2023, rendering the assurance of seed quality a focal point for the stable and synergistic development of agricultural systems.

Seed systems can be categorised into three types: formal, informal, and intermediate seed systems^8^. In China, the seed system predominantly operates within a formal framework, which is characterised by comprehensive government regulation, robust research and development, and the presence of large-scale seed companies. Consequently, this paper investigates the strategies for multilateral collaboration strategies in agricultural seed quality assurance within the formal system. In the production phase, seed companies, as suppliers, influenced by farmers' preferences, reputation, market competition, and government regulation, may opt to produce high-quality seeds. This strategy not only establishes a positive brand image but also secures trust from farmers and agribusinesses, thereby achieving sustainable development^9^. However, driven by complex economic conditions and market mechanisms, like cost–benefit analysis, insufficient market regulation, and information asymmetry, they might also choose to produce counterfeit and substandard seeds^10^. In the inspection phase, seed quality certification agencies, as regulatory agencies, in China, while regulated and supervised by government policies, operate as independent entities. Propelled by robust legal and market regulatory mechanisms along with a sense of professional ethics and responsibility, certification agencies may choose to resist rent-seeking behaviors^11^. Conversely, swayed by economic gains and individual utility maximisation, these agencies might adopt proactive rent-seeking strategies, engaging in informal collaborations with seed companies for additional financial returns^12^. In the feedback phase, epitomized by the adage, “a single grain sowed in spring yields thousands in autumn,” farmers, as the ultimate users and consumers of seeds, play a crucial role in assessing seed performance and influencing future seed improvements through their selections and feedback^5^. In instances of non-standard or other quality-related issues, farmers might opt for proactive rights protection to defend their legitimate interests, urging seed companies and certification agencies to prioritize seed quality, averting similar losses for other farmers. However, given the legal procedures, time, and financial costs entailed in rights protection, farmers might forego such actions. In the regulatory phase, the government, acting as a guardian in quality assurance^13^, might choose a stringent regulatory approach to propel seed companies and certification agencies towards enhanced standardization and professionalism. A lenient regulatory stance might be adopted to foster seed innovation and development, reduce operational costs for enterprises, and invigorate market dynamism and innovation^14^.

In agricultural systems, the stakeholders involved in seed quality assurance make decisions based on their interests and information^15^. To achieve the objectives of seed quality assurance, it is imperative to establish robust systems and mechanisms, guiding the parties towards collaborative relationships rather than adversarial ones, and collectively propelling the healthy and sustainable development of seeds^16^. This paper primarily addresses the following research questions: How can seed companies strike a balance between ensuring seed quality and reducing costs? How can seed quality certification agencies guarantee the fairness and accuracy of their evaluations? How can they build trust relationships with seed producers and farmers? How do farmers make decisions based on the cost of rights protection and compensation? How can the government establish an effective incentive and penalty mechanism to ensure that seed companies, certification agencies, and farmers adhere to good seed practices? To address the aforementioned issues, this paper initially employs a quadripartite evolutionary game theory to set up a mixed strategy game matrix based on the interest relations among seed companies, certification agencies, farmers, and the government in seed quality assurance, thereby establishing a game model. This analysis delves into the evolutionary stable strategies of each gaming subject and the impact mechanism of various factors on their strategy selections. Subsequently, utilizing Lyapunov’s First Method, the existence conditions for the combinations of evolutionary stable strategies are discerned. Lastly, a numerical simulation analysis is carried out using MATLAB 2023b to exhibit the evolutionary trajectories post alterations in influencing factors, and to validate the efficacy of the game analysis under different initial conditions. The study aims to contribute significantly towards augmenting the seed quality assurance mechanisms and propelling the sustainable progression of agricultural systems.

## Theoretical analysis

### Public governance theory

The Public Governance Theory, evolved from the amalgamation of political science theories, public administration theories, and economic analysis methods^17,18^, posits that governance entities encompass not only public governance institutions like governmental departments but also private institutions like enterprises and non-governmental organizations^19^. The essence of public governance lies in collaborative management^20,21^, where diverse governance entities partake and collaborate to augment the flexibility and effectiveness of management^22^. In scenarios where seed quality issues lead to reduced yields or total crop failure, solely relying on governmental departments for rectification might result in missing the sowing season, wasting land resources, and harming the direct interests and economic benefits of farmers and agri-businesses. Integrating Public Governance Theory into the seed quality assurance issue helps to elucidate the thought process behind quality assurance^23^. This theory accentuates the diversification of governance entities and the cooperative and consultative relations among them^24,25^. It guides the exploration of a cooperative win–win governance structure or model among seed companies, certification agencies, the general public, and regulatory departments. By doing so, it aids in constructing a comprehensive seed quality risk governance network, ensuring seed quality assurance. Through a public governance lens, this study aims to conceptualize and propose a robust governance structure encompassing multiple stakeholders to safeguard seed quality and, by extension, the broader agricultural system's sustainability and productivity.

### Evolutionary game theory

**Figure 1:**
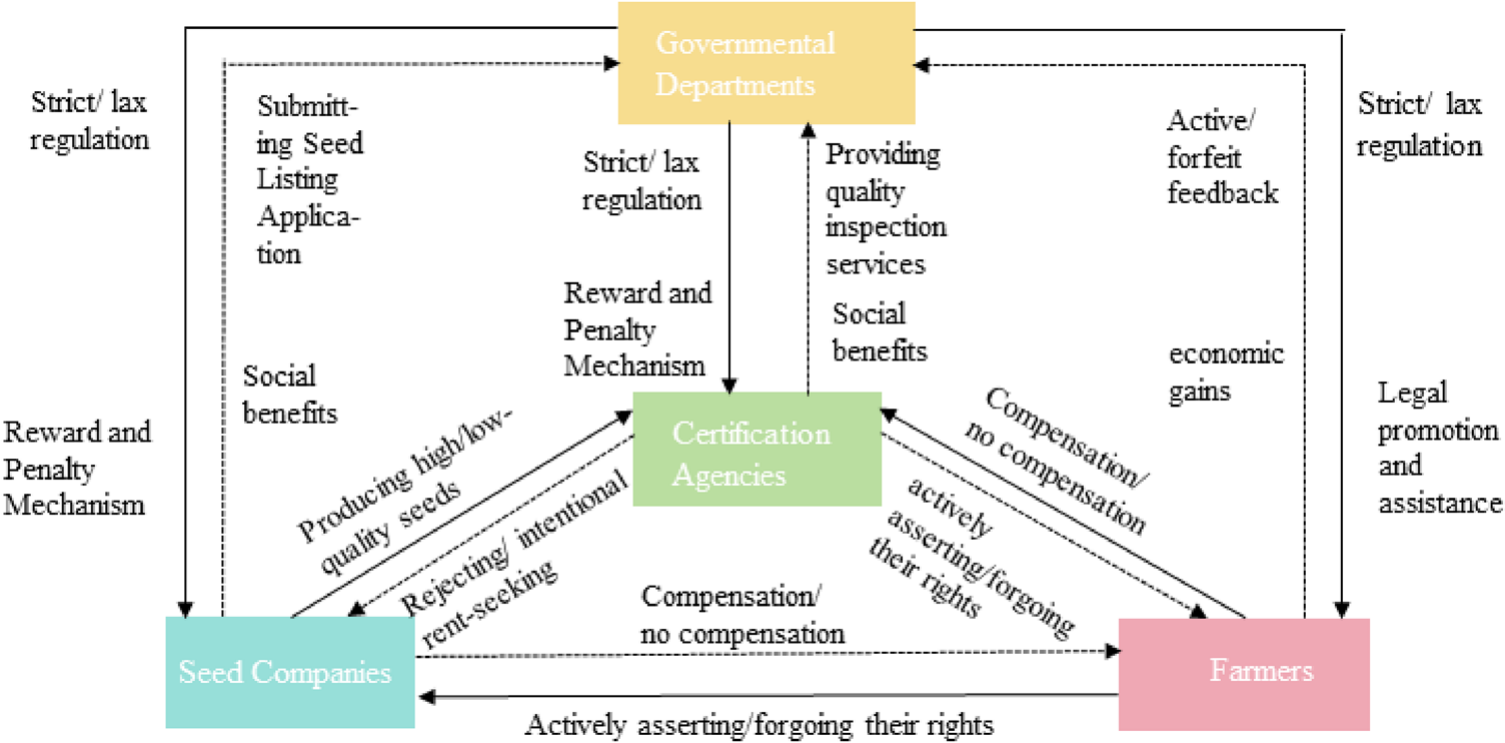
Logic relationship diagram of quadrilateral evolutionary game.

Evolutionary Game Theory revisits game equilibrium from an evolutionary perspective, relaxing the “complete rationality ” and assuming participants possess “bounded rationality,” providing a different analytical approach for Nash equilibrium and equilibrium selection^26^. Evolutionary game theory continues to be a hot topic in game theory research and is widely applied across various disciplines. The essence of seed quality assurance revolves around the game relationships that emerge concerning seed quality among seed companies, certification agencies, farmers, and the government. Seed is the “chip” of food security^27^, however, incidents of counterfeit and substandard seeds persist, the core issues remain unresolved largely due to the self-interest maximization objective during strategic selections by the responsible parties for seed quality assurance. The profit maximization by seed companies, rent-seeking behavior by certification agencies, and the cost–benefit analysis by farmers in rights protection contribute to this unresolved dilemma. Yet, external factors, such as varying regulatory strategies and incentive systems, can cause deviations in decisions. Stakeholders may adjust their game strategies based on different expected benefits, leading to new game equilibria^28^. The logical relationships among the gaming subjects are depicted in Fig. 1. Under varying degrees of influence from market forces and regulatory measures, seed companies, certification agencies, and farmers might choose different game strategies and take corresponding actions based on policy characteristics, regulatory intensity, self-profit, market reputation, inspection standards, and rights protection costs. And Governmental departments would evaluate the effectiveness of governance and regulations based on the actual situations and societal environment, analyze the trends in seed quality assurance, and adjust existing policies accordingly. From this perspective, the process of seed quality assurance can be viewed as an evolutionary game process among the responsible entities for seed quality assurance. This theoretical approach provides a structured framework to analyze and devise strategic interventions for enhancing seed quality assurance in agricultural systems.

## Model assumptions and construction

### Model assumptions

#### Assumption 1

In this model, four players are assumed: seed companies (Participant 1), seed quality certification agencies (Participant 2), farmers (Participant 3), and governmental departments (Participant 4). It’s posited that all participating entities possess bounded rationality and adhere to the principle of profit or utility maximization, with their strategic choices evolving over time towards optimal strategies.

#### Assumption 2

: The strategy space of seed companies $\alpha$ = (${\alpha }_{1}$, ${\alpha }_{2}$**) = **(produce high-quality seeds, produce fake and shoddy seeds), and with probability $x$ produces high-quality seeds, probability $1-x$ produces fake and shoddy seeds; the strategy space of seed quality certification agencies $\beta$** = **(${\beta }_{1}$, ${\beta }_{2}$) = (refuse to rent-seeking, engage to rent-seeking), the probability of its choice ${\beta }_{1}$ is y, and the probability of choosing ${\beta }_{2}$ is $1-y$; the strategy space of farmers $\gamma$** = **(${\gamma }_{1}$, ${\gamma }_{2}$) = (actively seek rights, give up the rights), and with probability $z$ chooses to actively seek the right, and the probability of $1-z$ to give up the right; the strategy space of the governmental departments $\delta =({\delta }_{1}$, ${\delta }_{2}$) = (strict regulation, lax regulation), and with probability $u$ chooses to strict supervise, and with probability $1-u$ chooses to lax regulation.

#### Assumption 3

The sales revenue of the seeds is denoted as ${R}_{p}$. The cost incurred by the seed company for producing high-quality seeds is represented as ${C}_{ph}$, whereas the cost for producing inferior seeds is denoted as ${C}_{pl}$, with ${C}_{ph}>{C}_{pl}$. When high-quality seeds are produced, they pass the quality inspection; conversely, when inferior seeds are produced, the company might opt to rent-seek from the certification agencies to secure market entry approval, with the rent-seeking cost represented as ${B}_{pr}$. Furthermore, the speculative behavior associated with the production of inferior seeds incurs a speculative cost, ${C}_{ps}$, which primarily include costs related to the falsification of seed vitality, moisture content, and germination rates, as well as expenses incurred from fraudulent advertising of seeds claimed to have disease resistance, strong adaptability, high yields, and enhanced nutritional values. The inferior seeds result in economic losses for the farmers, and during the process of their rights protection, compensation, ${M}_{p}$, needs to be provided to the farmers.

#### Assumption 4

The government establishes seed quality standards and oversees their overall implementation. Certification agencies assess and certify seed quality based on the standards set by the government. Seeds are permitted to enter the market for sale only after passing the inspection conducted by a third-party certification agencies. The revenue from inspection for the certification agencies is denoted as ${R}_{q}$, with the inspection cost represented as ${C}_{q}$. When inferior seeds are produced by the seed company, if the certification agencies refuses to engage in rent-seeking, the seeds fail the inspection and are not allowed into the market. Conversely, if the certification agencies is inclined towards rent-seeking, it engages in rent-seeking behavior with the seed company, assisting the inferior seeds in obtaining market entry approval. The revenue from rent-seeking for the certification agencies is denoted as ${R}_{qs}$, where ${R}_{qs}={B}_{pr}$, and the rent-seeking cost, ${C}_{qs}$, primarily encompasses expenses for fabricating basic information, production processes, and issuing false inspection reports. When the certification agencies engage in rent-seeking, aiding the seed company in getting market entry approval for the inferior seeds, it becomes liable in cases where farmers seek rights protection, bearing joint liability and providing compensation,${M}_{q}$.

#### Assumption 5

Farmers earn income, ${R}_{f}$, from planting high-quality seeds and harvesting them abundantly, with a cost of ${C}_{f}$, where ${C}_{f}={R}_{p}$. During the period of cultivation with inferior seeds, farmers carry out irrigation and fertilization. Yet, the low-quality seeds result in a reduced yield or even a total crop failure for the season, incurring a loss to the farmers denoted as ${H}_{f}$. Upon discovering quality issues with the seeds, timely rights protection actions by farmers can recuperate losses ${M}_{p}+{M}_{q}$. However, given the generally weak legal awareness among some farmers, they might opt for a quiet settlement when confronted with inferior seeds. To mitigate losses for other farmers, the government encourages farmers to actively seek rights protection when they encounter inferior seeds, denoted as ${R}_{fg}$, with the cost of rights protection represented by ${C}_{b}$.

#### Assumption 6

In the scenario of stringent regulation by governmental departments, seed companies producing and selling inferior seeds are subjected to a fine denoted as ${F}_{s}$, while certification agencies with rent-seeking intentions are fined ${F}_{q}$. Conversely, seed companies producing and selling high-quality seeds are rewarded with ${R}_{pg}$​, and certification agencies refusing rent-seeking are rewarded with ${R}_{qg}$. Under a lenient regulatory approach, the governmental departments do not proactively acquire strategic choice information from seed companies and certification agencies, thus, no rewards or punishments are administered. The cost incurred by governmental departments for regulation is represented as ${C}_{g}$.

#### Assumption 7

High-quality seeds are conducive to enhancing crop resistance to diseases, and improving both the yield and quality of varieties, which in turn increases the economic benefits for farmers, enhances consumer satisfaction, and promotes economic development and social stability, bringing about a social benefit, ${R}_{g}$, for the government. When seed companies produce inferior seeds and engage in rent-seeking transactions with certification agencies, the market infiltration of inferior seeds jeopardizes food security and economic development. To maintain social stability and rectify the seed industry, the government incurs a cost denoted as ${D}_{g}$. When governmental departments adopt a lax strategy, and due to regulatory lapses, situations where inferior seeds infiltrate the market occur, the governmental departments will be held accountable by superior authorities and subjected to administrative penalties denoted as ${T}_{g}$.

### Model construction

Based on the aforementioned assumptions and parameter settings, a mixed strategy game matrix involving seed companies, certification agencies, farmers, and governmental departments can be obtained, as shown in Table 1.

**Table 1:** Mixed strategy game matrix.

Strategy selection		Certification agencies	Governmental departments			
			Strict regulation ($u$)		Lax regulation($1-u$)	
			Farmers			
			Actively asserting their rights (${\text{z}}$)	Forgoing their rights($1-z$)	Actively asserting their rights ($z$)	Forgoing their rights ($1-z$)
Seed companies	Producing high-quality seeds $x$	Rejecting rent-seeking $y$	${R}_{p}-{C}_{ph}+{R}_{pg}$, ${R}_{q}-{C}_{q}+{R}_{qg}$, ${R}_{f}-{C}_{f}$, ${R}_{g}-{C}_{g}-{R}_{pg}-{R}_{qg}$	$-{C}_{ph}+{R}_{pg}$, ${R}_{q}-{C}_{q}+{R}_{qg}$, ${R}_{f}-{C}_{f}$, ${R}_{g}-{C}_{g}-{R}_{pg}-{R}_{qg}$	${R}_{p}-{C}_{ph}$, ${R}_{q}-{C}_{q}$, ${R}_{f}-{C}_{f}$, ${R}_{g}-{C}_{g}$	${R}_{p}-{C}_{ph}$, ${R}_{q}-{C}_{q}$, ${R}_{f}-{C}_{f}$, ${R}_{g}-{C}_{g}$
		Intentional rent-seeking $1-y$	${R}_{p}-{C}_{ph}+{R}_{pg}$**,** ${R}_{q}-{C}_{q}-{F}_{q}$**,** ${R}_{f}-{C}_{f}$**,** ${R}_{g}-{C}_{g}-{R}_{pg}+{F}_{q}$	${R}_{p}-{C}_{ph}+{R}_{pg}$**,** ${R}_{q}-{C}_{q}-{F}_{q}$**,** ${R}_{f}-{C}_{f}$**,** ${R}_{g}-{C}_{g}-{R}_{pg}+{F}_{q}$	${R}_{p}-{C}_{ph}$**,** ${R}_{q}-{C}_{q}$**,** ${R}_{f}-{C}_{f}$**,** ${R}_{g}-{C}_{g}$	${R}_{p}-{C}_{ph}$**,** ${R}_{q}-{C}_{q}$**,** ${R}_{f}-{C}_{f}$**,** ${R}_{g}-{C}_{g}$
	Producing low-quality seeds $1- x$	Refusal to seek rent $y$	$-{C}_{pl}-{C}_{ps}-{F}_{s}$, ${R}_{q}-{C}_{q}+{R}_{qg}$, 0, ${F}_{s}-{C}_{g}-{R}_{qg}$	$-{C}_{pl}-{C}_{ps}-{F}_{s}$, ${R}_{q}-{C}_{q}+{R}_{qg}$, 0, ${F}_{s}-{C}_{g}-{R}_{qg}$	$-{C}_{pl}-{C}_{ps}$, ${R}_{q}-{C}_{q}$, 0,$-{C}_{g}$	$-{C}_{pl}-{C}_{ps}$, ${R}_{q}-{C}_{q}$, 0,$-{C}_{g}$
		Intentional rent-seeking $1-y$	${R}_{p}-{C}_{pl}-{B}_{pr}-{C}_{ps}-{F}_{s}-{M}_{p}$, ${R}_{q}-{C}_{q}-{F}_{q}+{R}_{qs}-{C}_{ps}-{M}_{q}$, $-{C}_{f}-{H}_{f}+{M}_{p}+{M}_{q}+{R}_{fg}-{C}_{b}$, ${F}_{s}+{F}_{q}-{C}_{g}-{D}_{g}-{R}_{fg}$	${R}_{p}-{C}_{pl}-{B}_{pr}-{C}_{ps}-{F}_{s}$, ${R}_{q}-{C}_{q}-{F}_{q}+{R}_{qs}-{C}_{ps}$, $-{C}_{f}-{H}_{f}$, ${F}_{s}+{F}_{q}-{C}_{g}-{D}_{g}$	${R}_{p}-{C}_{pl}-{B}_{pr}-{C}_{ps}-{M}_{p}$, ${R}_{q}-{C}_{q}+{R}_{qs}-{C}_{ps}-{M}_{q}$, $-{C}_{f}-{H}_{f}+{M}_{p}+{M}_{q}-{C}_{b}$, $-{D}_{g}-{T}_{g}-{C}_{g}$	${R}_{p}-{C}_{pl}-{B}_{pr}-{C}_{ps}$, ${R}_{q}-{C}_{q}+{R}_{qs}-{C}_{ps}$, $-{C}_{f}-{H}_{f}$, $-{D}_{g}-{T}_{g}-{C}_{g}$

## Results and analysis

### Analysis on strategic stability of the game subjects

#### Strategic stability in seed companies production

The expected earnings from seed companies producing either high-quality or inferior seeds, along with the average expected earnings (${E}_{11}, {E}_{12}, \overline{{E }_{1}}$), are as follows:

1 $$\left\{\begin{array}{l}{E}_{11}={R}_{p}-{C}_{ph}+u{R}_{pg}\\ {E}_{12}={R}_{p}-{C}_{pl}-{C}_{ps}-{B}_{pr}+y{B}_{pr}-{uF}_{s}-{zM}_{p}-y{R}_{p}+yz{M}_{p}\\ \overline{{E }_{1}} =x{E}_{11}+\left(1-x\right){E}_{12}\end{array}\right.$$

The replicator dynamic equation for the production strategy of seed companies is:

2 $$F\left(x\right)=\frac{dx}{dt} =x\left({E}_{11}-\overline{{E }_{1}}\right) =x(x-1)({C}_{ph}-{B}_{pr}-{C}_{pl}-{C}_{ps}+y{B}_{pr}-{uF}_{s}- u{R}_{pg}-{zM}_{p}-y{R}_{p}+yz{M}_{p})$$

The first derivative of $F(x)$ is:

3 $$d(F(x))/dx=\left(2x-1\right)({C}_{ph}-{B}_{pr}-{C}_{pl} -{C}_{ps}+y{B}_{pr}-{uF}_{s}-u{R}_{pg}-{zM}_{p}-y{R}_{p}+yz{M}_{p})$$

Based on the stability theorem of differential equations, it is known that the strategy of seed companies choosing to produce high-quality seeds must satisfy the following condition for a stable state:$F\left(x\right)=0\,\, and\,\, d(F(x))/dx<0$

##### Proposition 1

*When* $y>{y}_{1}$, $z<{z}_{1}$, *and* $u<{u}_{1}$, *the stable strategy for seed companies is to produce high-quality seeds; when and* $y<{y}_{1}$, $z>{z}_{1}$, *and* $u>{u}_{1}$, *the stable strategy is to produce inferior seeds; when* $y={y}_{1}$, $z={z}_{1}$, *and* $u={u}_{1}$, *the seed companies cannot determine their stable strategy. Where the threshold values are*:

4 $$\left\{\begin{array}{l}{y}_{1}=({C}_{ph}-{B}_{pr}-{C}_{pl} -{C}_{ps}- {uF}_{s}- {zM}_{p}- u{R}_{pg}/({R}_{p}-{zM}_{p}-{B}_{pr})\\ {z}_{1}=( {C}_{ph}-{B}_{pr}-{C}_{pl} -{C}_{ps}+y{B}_{pr}-{uF}_{s}-u{R}_{pg}- y{R}_{p} )/({M}_{p}-y{M}_{p})\\ {u}_{1}=( {C}_{ph}-{B}_{pr}-{C}_{pl} -{C}_{ps}+y{B}_{pr}- {zM}_{p}- y{R}_{p}+ yz{M}_{p})/({F}_{s}+{R}_{pg})\end{array}\right.$$

##### Proof

Let $$G\left(y\right)={C}_{ph}-{B}_{pr}-{C}_{pl}-{C}_{ps}+y{B}_{pr}-{uF}_{s}- u{R}_{pg}-{zM}_{p}-y{R}_{p}+yz{M}_{p}$$.

Setting $F\left(x\right)=0$, results in $x=0$ or $x=1$ or $G\left(y\right)=0$. $\partial G\left(y\right)/\partial y={B}_{pr}+{zM}_{p}-{R}_{p}<0$, indicating that $G\left(y\right)$ is a decreasing function of $y$, When $y>{y}_{1}$, $G\left(y\right)<0$, and only when $x=1$ does it satisfy the condition for strategy stability $F\left(x\right)=0$ and $d(F(x))/dx<0$ get satisfied. Similarly, when $<{y}_{1}$, $G\left(y\right)>0$, and only when $x=0$ does it satisfy the condition for strategy stability $F\left(x\right)=0$ and $d(F(x))/dx<0$ get satisfied. When $y={y}_{1}$, $G\left(y\right)=0$, and $d(F(x))/dx\equiv 0$, the seed companies cannot determine its stable strategy. The impact of threshold values ${z}_{1}$ and ${u}_{1}$ on strategy stability can be demonstrated analogously.

**Figure 2:**
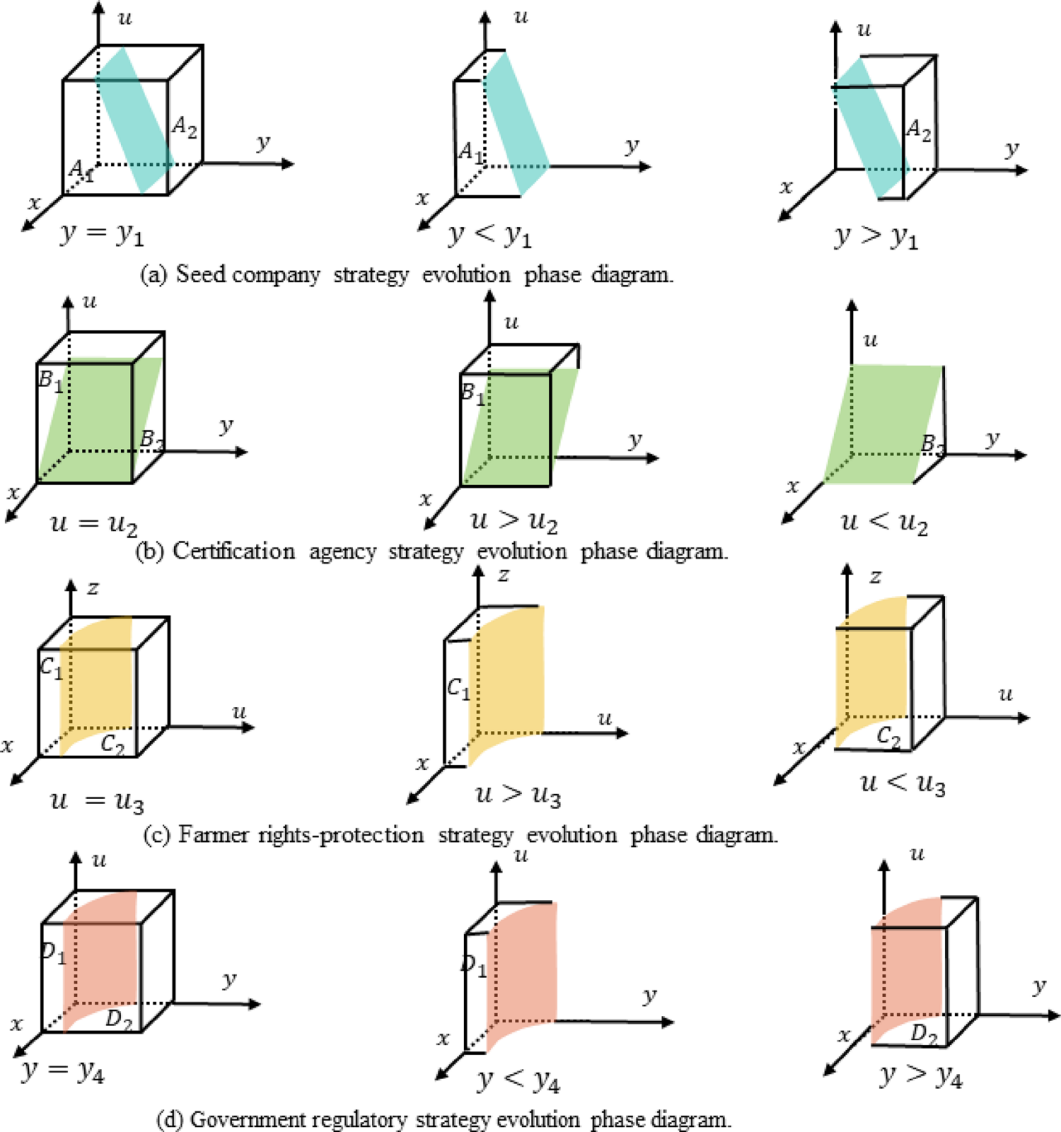
Phase diagram of evolutionary strategies among all parties.

According to Proposition 1, The phase diagram of the seed companies' strategic choice is shown in Fig. 2a, indicating that the probability of seed companies choosing to produce inferior seeds corresponds to the volume ${V}_{A1}$ of region ${A}_{1}$, and the probability of choosing to produce high-quality seeds corresponds to the volume ${V}_{A2}$​ of region ${A}_{2}$​. Upon calculation, it is found:

5 $${V}_{A1}={\int }_{0}^{1}{\int }_{0}^{1}{y}_{1}dxdu= \left({C}_{ph}-{B}_{pr}-{C}_{pl} -{C}_{ps}-\frac{{F}_{s}}{2}-\frac{{R}_{pg}}{2}-{zM}_{p}\right)({R}_{p}-{zM}_{p}-{B}_{pr})$$

6 $${V}_{A2}=1-{V}_{A1}$$

##### Corollary 1

*The probability of seed companies producing high-quality products is positively correlated with speculative cost* (${C}_{ps}$), *the cost of inferior seeds* (${C}_{pl}$), *government rewards* (${R}_{pg}$), *and government fines* (${F}_{s}$), *and negatively correlated with production cost* (${C}_{ph}$).

##### Proof

Based on the expression ${V}_{A2}$ for the probability of seed companies producing high-quality seeds, we calculate the first-order partial derivatives with respect to each influencing factor, yielding:

$$\partial {V}_{A2}/\partial {R}_{pg}>0, \partial {V}_{A2}/\partial {C}_{ph}<0, \partial {V}_{A2}/\partial {C}_{pl}>0, \partial {V}_{A2}/\partial {C}_{ps}>0, \partial {V}_{A2}/\partial {F}_{s}>0, \partial {V}_{A2}/\partial \left({C}_{ph}-{C}_{pl}\right)<0.$$

#### Strategic stability in rent-seeking by certification agencies

The expected earnings from either refusal or intention of rent-seeking by the certification agencies, along with the average expected earnings (${E}_{21}$, ${E}_{22}$, $\overline{{E }_{2}}$) are as follows:

7 $$\left\{\begin{array}{l}{E}_{21}={{\text{R}}}_{q}-{C}_{q}+{uR}_{qg}\\ {E}_{22}={R}_{q}-{C}_{qs}-{C}_{q}+{R}_{qs}+x{C}_{qs}-z{M}_{q}-x{R}_{qs}+xz{M}_{q}\\ {E}_{22}={R}_{q}-{C}_{qs}-{C}_{q}+{R}_{qs}+x{C}_{qs}-z{M}_{q}-x{R}_{qs}+xz{M}_{q}\end{array}\right.$$

The replicator dynamic equation for certification agencies is:

8 $$F\left(y\right)=dy/dt=y\left({E}_{21}-\overline{{E }_{2}}\right)=y(y-1)({R}_{qs}-{C}_{qs}-u{F}_{q }+x{C}_{qs}-z{M}_{q}-{uR}_{qg}-x{R}_{qs}+xz{M}_{q})$$

The first derivative of $F(y)$ is:

9 $$d\left(F\left(y\right)\right)/y=\left(2y-1\right)\left({R}_{qs}-{C}_{qs}-u{F}_{q }+x{C}_{qs}-z{M}_{q}-{uR}_{qg}-x{R}_{qs}+xz{M}_{q}\right)$$

Based on the stability theorem of differential equations, it is known that for the strategy of rent-seeking refusal by the certification agencies to be in a stable state, it must satisfy:$F\left(y\right)=0$ and $d(F(y))/dy<0$.

##### Proposition 2

*When* $x>{x}_{2}$, ${z>z}_{2}$, *and* $u>{u}_{2}$, *the stable strategy for the certification agencies is to refuse rent-seeking; when* $u<{u}_{2}$, $x<{x}_{2}$, *and* ${z<z}_{2}$, *the stable strategy for the certification agencies is to intend rent-seeking; when* $u={u}_{2}$, $x={x}_{2}$, *and* ${z=z}_{2}$, *the certification agencies cannot determine their stable strategy. Threshold values*:

10 $$\left\{\begin{array}{l}{u}_{2}=({R}_{qs}-{C}_{qs}+x{C}_{qs} -z{M}_{q}- x{R}_{qs}+xz{M}_{q})/({F}_{q }+{R}_{qg})\\ {x}_{2}=({R}_{qs}-{C}_{qs} - u{F}_{q } - z{M}_{q}- {uR}_{qg})/({R}_{qs}-Cqs-z{M}_{q})\\ {z}_{2}=({R}_{qs}-{C}_{qs} -u{F}_{q } +xCqs- z{M}_{q}-{uR}_{qg}-x{R}_{qs} +xz{M}_{q} )/({M}_{q}-x{M}_{q})\end{array}\right.$$

##### Proof

Let $H(u)={R}_{qs}-{C}_{qs}-u{F}_{q }+x{C}_{qs}-z{M}_{q}-{uR}_{qg}-x{R}_{qs}+xz{M}_{q}$. Setting $F\left(y\right)=0$, yields $y=0$ or $y=1$ or $H(u)=0$. Given that $\partial H(u)/\partial u=-{R}_{qg}-{F}_{q}<0$, it is evident that $H(u)$ is a decreasing function of $u$. When $u>{u}_{2}$, $H(u)<0$, and only when $y=1$ does it satisfy the condition for strategy stability $F\left(y\right)=0$ and $d(F(y))/dy<0$. Similarly, when $<{u}_{2}$, $H(u)>0$, and only when $y=0$ does it satisfy the condition for strategy stability $\left(y\right)=0$ and $d(F(y))/dy<0$. When $\left(y\right)=0$ and $(F(y))/dy<0$, $H(u)=0$, and $d(F(y))/dy\equiv 0$, the certification agencies are unable to determine their stable strategy. The impact of threshold values ${x}_{2}$ and ${z}_{2}$ on strategy stability can be demonstrated analogously.

According to Proposition 2, The phase diagram of the strategy choices by the certification agencies is shown in Fig. 2b, illustrating that the probability of refusing rent-seeking is represented by the volume ${V}_{B1}$ of area ${B}_{1}$, while the probability of intending to seek rent is represented by the volume ${V}_{B2}$ of area ${B}_{2}$. Through calculation, it is obtained that:

11 $${V}_{B2}={\int }_{0}^{1}{\int }_{0}^{1}{u}_{2}dxdy=-({C}_{qs}-{R}_{qs} + z{M}_{q} )/(2({F}_{q }+{R}_{qg}))$$

12 $${V}_{B1}=1-{V}_{B2}$$

##### Corollary 2

*The probability of seed quality certification agencies refusing rent-seeking is positively correlated with rent-seeking cost* (${C}_{qs}$) *and illegal compensation* (${M}_{q}$), *and negatively correlated with rent-seeking revenue* (${R}_{qs}$).

##### Proof

By deriving the first-order partial derivatives from the expression for the probability ${V}_{B1}$ of certification agencies refusing rent-seeking for each influencing factor, it is obtained: ${\partial {V}_{B1}}/{\partial {R}_{qs}}<{0, \partial {V}_{B1}}/{{\partial C}_{qs}}>0,\partial {V}_{B1}/{\partial M}_{q}>0.$

#### Strategic stability in farmers' rights protection

The expected earnings from either active rights protection or forfeiture of rights protection by the farmers, along with the average expected earnings (${E}_{31}$, ${E}_{32}$, $\overline{{E }_{3}}$), are as follows:

13 $$\left\{\begin{array}{l}{E}_{31}=\left(u - 1\right)\left(x - 1\right)\left(y - 1\right)\left({C}_{b}+{C}_{f}+{H}_{f}-{M}_{p}-{M}_{q}\right)-x\left( {C}_{f}-{R}_{f}\right)\\ -u\left(x - 1\right)\left(y - 1\right)({C}_{b}+{C}_{f}+{H}_{f}-{M}_{p}-{M}_{q}-{R}_{fg})\\ {E}_{32}=- x({C}_{f}-{R}_{f})-(x-1)(y-1)({C}_{f}+{H}_{f})\\ \overline{{E }_{3}}= z{E}_{31}+\left(1-z\right){E}_{32}\end{array}\right.$$

The replicator dynamic equation for the farmers' rights protection strategy is as follows:

14 $$F\left(z\right)=dz/dt=z\left({E}_{31}-\overline{{E }_{3}}\right) =z(z-1)(-(x - 1)(y - 1)({M}_{p}-{{\text{C}}}_{b}+{M}_{q}+u{R}_{fg}))$$

The first derivative of $F(z)$ is:

15 $$d\left(F\left(z\right)\right)/dz=(2z-1) (-(x - 1)(y - 1)({M}_{p}-{{\text{C}}}_{b}+{M}_{q}+u{R}_{fg}))$$

Based on the stability theorem of differential equation, it is imperative that the strategy of farmers actively seeking rights must satisfy the following conditions:$F\left(z\right)=0$ and $d(F(z))/dz<0$

##### Proposition 3

*When* $x>{x}_{3}$, ${y>y}_{3}$​, *and* $u>{u}_{3}$, *the stable strategy for farmers is to actively seek rights. Conversely, when* $x<{x}_{3}$, ${y<y}_{3}$​, *and* $u<{u}_{3}$, *the stable strategy chosen by farmers is to forgo rights. However, when* $x={x}_{3}$, ${y=y}_{3}$​, *and* $u={u}_{3}$, *farmers are unable to ascertain their stable strategy. Threshold values are as follows*:

16 $$\left\{\begin{array}{l}{u}_{3}=({{\text{C}}}_{b}-{M}_{p}-{M}_{q}-x {{\text{C}}}_{b}-y{{\text{C}}}_{b}+x{M}_{p}+x{M}_{q}+y{M}_{p}+y{M}_{q}+xy{{\text{C}}}_{b}\\ -xy{M}_{p}- xy {M}_{q})/( {R}_{fg}-x{R}_{fg}-y{R}_{fg}+xy{R}_{fg})\\ {x}_{3}=\left({{\text{C}}}_{b}-{M}_{p}-{M}_{q}- y{{\text{C}}}_{b} +y{M}_{p} +y{M}_{q} - u{R}_{fg} + uy{R}_{fg} \right)\\ /({{\text{C}}}_{b}- {M}_{p}- {M}_{q}-y{{\text{C}}}_{b}+y{M}_{p}+y{M}_{q}-u{R}_{fg}+uy{R}_{fg})\\ {y}_{3}=({{\text{C}}}_{b}-{M}_{p}-{M}_{q}-xCb-y{{\text{C}}}_{b}+x{M}_{p}+x{M}_{q}+y{M}_{p}\\ +y{M}_{q}-u{R}_{fg}+xy{{\text{C}}}_{b}-xy{M}_{p}-xy{M}_{q}+ux{R}_{fg}+uy{R}_{fg}-uxy{R}_{fg})\\ /( {{\text{C}}}_{b}-{M}_{p}-{M}_{q}-x{{\text{C}}}_{b}+x{M}_{p}+x{M}_{q}-u{R}_{fg}-xu{R}_{fg})\end{array}\right.$$

##### Proof

Let $I\left(u\right)=-(x - 1)(y - 1)({M}_{p}-{{\text{C}}}_{b}+{M}_{q}+u{R}_{fg})$. Setting $F\left(z\right)=0$, yields $z=0$ or $z=1$ or $I\left(u\right)=0$. Since $\partial I\left(u\right)/\partial u=-(x - 1)(y - 1){R}_{fg}<0$, it follows that $I\left(u\right)$ is a decreasing function of $u$. When $u>{u}_{3}$, $I\left(u\right)<0$, and only when $y=1$ is the stable strategy condition $F\left(y\right)=0$ and $d(F(y))/dy<0$ satisfied. Similarly, when $<{u}_{3}$, $I\left(u\right)>0$, and only when $y=0$ is the stable strategy condition $F\left(y\right)=0$ and $d(F(y))/dy<0$ satisfied. When $u={u}_{3}$, $I\left(u\right)=0$, $d(F(y))/dy\equiv 0$, the certification agencies cannot determine its stable strategy. The impact of threshold values ${x}_{3}$ and ${y}_{3}$ on strategy stability can be proven analogously.

 Based on Proposition 3, the phase diagram of farmers' strategy selection is shown in Fig. 2c, indicating that the probability of farmers foregoing their rights is represented by the volume of ${V}_{c1}$ in region ${C}_{1}$, while the probability of farmers actively asserting their rights is represented by the volume of ${V}_{c2}$ in region ${C}_{2}$. The calculations are as follows:

17 $${V}_{c1}={\int }_{0}^{1}{\int }_{0}^{1}{u}_{3}dxdz=-({M}_{p}-{{\text{C}}}_{b}+{M}_{q})/{R}_{fg}$$

18 $${V}_{c2}=1-{V}_{c1}$$

##### Corollary 3

*The probability of farmers actively asserting their rights is positively correlated with the compensation received from seed companies and certification agencies* (${M}_{p}\mathrm{ and }{M}_{q}$), *and is negatively correlated with the cost of rights assertion* (${C}_{b}$).

##### Proof

By taking the partial derivatives of the expression for the probability of farmers actively asserting their rights (${V}_{c2}$) with respect to each influencing factor, the following is obtained: ${\partial {V}_{c2}}/{\partial {M}_{p}}={1}/{{R}_{fg}}>{0, \partial {V}_{c2}}/{\partial {C}_{b}}=-{1}/{{R}_{fg}}<0,\partial {V}_{c2}/{\partial M}_{q}=1/{R}_{fg}>0.$

#### Strategic stability in government regulation

The expected earnings from either strict or lax regulation by the government, along with the average expected earnings (${E}_{41}$, ${E}_{42}$, $\overline{{E }_{4}}$) are as follows:

19 $$\left\{\begin{array}{l}{E}_{41}=y\left(x - 1\right)\left({{\text{C}}}_{g}-{F}_{s}+{R}_{qg}\right)-xy\left({{\text{C}}}_{g}-{R}_{g }+ {R}_{pg }+ {R}_{qg }\right)+x\left(y-1\right)\left({{\text{C}}}_{g}-{F}_{q}-{R}_{g }+ {R}_{pg }\right)\\ -z(x-1)(y-1)({{\text{C}}}_{g}+{D}_{g }-{F}_{q}-{F}_{s}+{R}_{fg})+(x-1)(y-1)(z-1)({{\text{C}}}_{g}+{D}_{g }-{F}_{q}-{F}_{s})\\ {E}_{42}=y\left(x-1\right){{\text{C}}}_{g}-x\left({{\text{C}}}_{g}-{R}_{g }\right)+\left(x-1\right)\left(y-1\right)\left(z-1\right)\left({{\text{C}}}_{g}+{D}_{g }+{T}_{g}\right)\\ -z(x-1)(y-1)({{\text{C}}}_{g}+{D}_{g }+{T}_{g})\\ \overline{{E }_{4}}= u{E}_{41}+\left(1-u\right){E}_{42}\end{array}\right.$$

The replicator dynamic equation for government regulation is:

20 $$F\left(u\right)=du/dt=u(u-1)(x{F}_{s}-{F}_{s}-{T}_{g}-{F}_{q}+y{F}_{q}+x{R}_{pg }+y{R}_{qg}+z{R}_{fg}+{ xT}_{g}+y{T}_{g}- xz {R}_{fg}-yz{R}_{fg}-xy{T}_{g}+xyz{R}_{fg})$$

The first derivative of $F(u)$ is:

21 $$d\left(F\left(u\right)\right)/du=(2u-1)(x{F}_{s}-{F}_{s}-{T}_{g}-{F}_{q}+y{F}_{q}+x{R}_{pg }+y{R}_{qg}+z{R}_{fg}+{ xT}_{g}+y{T}_{g}- xz{R}_{fg}-yz{R}_{fg}-xy{T}_{g}+xyz{R}_{fg})$$

According to the stability theorem of differential equations, the strategy of strict regulation chosen by the government must satisfy:$F\left(u\right)=0$ and $d(F(u))/du<0$.

##### Proposition 4

*When* $x<{x}_{4}$, ${y<y}_{4}$, $z<{z}_{4}$, *the stable strategy for the governmental departments is strict regulation; when* $x>{x}_{4}$, ${y>y}_{4}$, $z>{z}_{4}$, *the stable strategy for the governmental departments is lax regulation; when* $x={x}_{4}$, ${y=y}_{4}$, $z={z}_{4}$, *the governmental departments cannot determine its stable strategy. Threshold values*:

22 $$\left\{\begin{array}{l}{x}_{4}=(-{F}_{s}-{T}_{g}-{F}_{q}+y{F}_{q}+y{R}_{qg}+z{R}_{fg}+y{T}_{g}-yz{R}_{fg})/(-{F}_{s}-{R}_{pg }-{T}_{g}+z{R}_{fg}+y{T}_{g}-yz{R}_{fg})\\ {{\text{y}}}_{4}=(x{F}_{s}-{F}_{s}-{T}_{g}-{F}_{q}+x{R}_{pg }+z{R}_{fg}+{ xT}_{g}-xz{R}_{fg})/(-{F}_{q}-{R}_{qg}-{T}_{g}+z{R}_{fg}+x{T}_{g}-xz{R}_{fg})\\ {z}_{4}=({F}_{s}+{T}_{g}+{F}_{q}-y{F}_{q}-x{R}_{pg }-y{R}_{qg}-{ xT}_{g}-y{T}_{g}+xy{T}_{g}-x{F}_{s})/({R}_{fg}-x{R}_{fg}-y{R}_{fg}+xy{R}_{fg})\end{array}\right.$$

##### Proof

Let $J\left(y\right)=(x{F}_{s}-{F}_{s}-{T}_{g}-{F}_{q}+y{F}_{q}+x{R}_{pg }+y{R}_{qg}+z{R}_{fg}+{ xT}_{g}+y{T}_{g}-xz {R}_{fg}-yz{R}_{fg}-xy{T}_{g}+xyz{R}_{fg})$. Setting $F\left(u\right)=0$, it yields $u=0$ or $u=1$ or $J\left(y\right)=0$. $\partial J\left(y\right)/\partial y={F}_{q}+{R}_{qg}+{T}_{g}-z{R}_{fg}-x{T}_{g}+xz{R}_{fg}>0$, Since $Tg>R$, it follows that $J\left(y\right)$ is an increasing function of $y$. When ${y<y}_{4}$, $J\left(y\right)<0$, and only when $u=1$ is the strategic stability condition $F\left(u\right)=0$ and $d(F(u))/du<0$ satisfied. Similarly, when ${y>y}_{4}$, $J\left(y\right)>0$, and only when $u=0$ is the strategic stability condition $F\left(u\right)=0$ and $d(F(u))/du<0$ satisfied. When ${y=y}_{4}$, $J\left(y\right)=0$, $d(F(u))/du\equiv 0$, the governmental departments cannot determine its stable strategy. The effects of the threshold values ${x}_{4}$ and ${z}_{4}$ on strategic stability can be proved in a similar manner.

Based on Proposition 4, the phase diagram of the strategic choice of the governmental departments is shown in Fig. 2d. It demonstrates that the probability of the governmental departments choosing strict regulation is represented by the volume of ${D}_{1}$, denoted as ${V}_{D1}$, while the probability of choosing loose regulation is represented by the volume of ${D}_{2}$, denoted as ${V}_{D2}$. The calculations yield:

23 $${V}_{D1}={\int }_{0}^{1}{\int }_{0}^{1}{z}_{4}dxdu=({F}_{q}+{R}_{qg}+{T}_{g}/2)/{R}_{fg}+({R}_{pg }/2-{F}_{s}/2 +{R}_{qg})/((y - 1){R}_{fg})$$

24 $${V}_{D2}=1-{V}_{D1}$$

##### Corollary 4

*The probability of the governmental departments choosing strict regulation is positively correlated with the fines imposed on seed companies for producing inferior seeds* (${F}_{s}$), *fines on certification agencies for active rent-seeking* (${F}_{q}$), *and higher-level accountability* (${T}_{g}$), *while it is negatively correlated with the rewards given based on the production of high-quality seeds* (${R}_{pg}$).

##### Proof

 By deriving the first-order partial derivatives from the expression ${V}_{D2}$ for the probability of the governmental departments choosing strict regulation, we obtain:$\partial {V}_{D1}/\partial {F}_{s}=-1/\left(2{\left(y-1\right)R}_{fg}\right)>0, \partial {V}_{D1}/\partial {F}_{q}=1/{R}_{fg}>0, \partial {V}_{D1}/\partial {R}_{pg}= 1/(2(\mathrm{y }- 1){R}_{fg}<0, \partial {V}_{D1}/\partial {T}_{g}>0$

#### Equilibrium points in the four-party evolutionary game system

In the dynamic system of game interactions among seed companies, certification agencies, farmers, and government, the stability of strategy selection can be determined using Lyapunov's First Method. The Jacobian matrix of this four-party evolutionary game in this study is:

$$J=\left[\begin{array}{cc}\begin{array}{c}\partial F\left(x\right)/\partial x\\ \partial F(y)/\partial x\\ \partial F\left(z\right)/\partial x\end{array}& \begin{array}{c}\begin{array}{ccc}\partial F\left(x\right)/\partial y& \partial F\left(x\right)/\partial z& \partial F\left(x\right)/\partial u\end{array}\\ \begin{array}{ccc}\partial F(y)/\partial y& \partial F(y)/\partial z& \partial F(y)/\partial u\end{array}\\ \partial \begin{array}{ccc}F\left(z\right)/\partial y& \partial F\left(z\right)/\partial z& \partial F\left(z\right)/\partial u\end{array}\end{array}\\ \partial F\left(u\right)/\partial x& \begin{array}{ccc}\partial F\left(u\right)/\partial y& \partial F\left(u\right)/\partial z& \partial F\left(u\right)/\partial u\end{array}\end{array}\right]$$

Setting $F\left(x\right)$= $F(y)$= $F(z)$= $F\left(u\right)$=0, 43 sets of system equilibrium solutions are obtained. Among them, 16 sets of pure strategy equilibrium solutions are substituted into the Jacobian matrix to obtain 16 sets of matrix eigenvalues. As seen in Table 2, under the relevant conditions, there are two strategy combinations, (0, 0, 1, 1) and (0, 0, 1, 0), which belong to ESS points.

**Table 2:** Stability analysis of equilibrium points.

Equilibrium points	Jacobian matrix eigenvalues		Stability conclusions	Conditions
	${\lambda_1},\;{\lambda_2},\;{\lambda_3}$	Sign of the real part		
(1, 0, 0, 0)	0, 0, ${F}_{q}$ − ${R}_{pg}$, ${C}_{ph}$ − ${B}_{pr}$ − ${C}_{pl}$ − ${C}_{ps}$	(0, 0, ×, ×)	Uncertainty	–
(0, 0, 0, 0)	${C}_{qs}$ − ${R}_{qs}$, ${F}_{q}$+${F}_{s}$+${T}_{g}$, ${M}_{p}$ − ${C}_{b}$+${M}_{q}$,${B}_{pr}$ − ${C}_{ph}$+${C}_{pl}$+${C}_{ps}$	(−, +, ×, ×)	Unstable points	–
(0, 1, 0, 0)	0,${R}_{qs}$ − ${C}_{qs}$, ${F}_{s}$− ${R}_{qg}$, ${C}_{pl}$− ${C}_{ph}$+${C}_{ps}$+${R}_{p}$	(0, +, ×, ×)	Unstable points	–
(0, 0, 1, 0)	1/(${B}_{pr}$− ${C}_{ph}$+${C}_{pl}$+${C}_{ps}$+${M}_{p}$), − 1/(Cqs + ${M}_{q}$− ${R}_{qs}$), − 1/(${M}_{p}$− ${C}_{b}$+${M}_{q}$), 1/(${F}_{q}$+${F}_{s}$− ${R}_{fg}$+${T}_{g}$	(−, −, −, −)	ESS	①② ③④
(0, 0, 0, 1)	1/(${B}_{pr}$− ${C}_{ph}$+${C}_{pl}$+${C}_{ps}$+${F}_{s}$+ ${R}_{pg}$), 1/(${C}_{qs}$+${F}_{q}$+${R}_{qg}$− ${R}_{qs}$),1/(${M}_{p}$− ${C}_{b}$+${M}_{q}$+${R}_{fg}$), − 1/(${F}_{q}$ +${F}_{s}$+${T}_{g}$)	(×, ×, +, ×)	Unstable points	–
(1, 1, 0, 0)	0, 0,− ${R}_{pg}$− ${R}_{qg}$,${C}_{ph}$− ${C}_{pl}$− ${C}_{ps}$− ${R}_{p}$	(0, 0, −, ×)	Uncertainty	–
(1, 0, 1, 0)	0, 0, ${F}_{q}$− ${R}_{pg}$,${C}_{ph}$− ${B}_{pr}$− ${C}_{pl}$− ${C}_{ps}$− ${M}_{p}$	(0, 0, ×, ×)	Uncertainty	–
(0, 1, 1, 0)	0, ${F}_{s}$− Rqg, ${R}_{qs}$− Mq-Cqs, ${C}_{pl}$ − ${C}_{ph}$+${C}_{ps}$+ Rp	(0, 0, ×, ×)	Uncertainty	–
(1, 0, 0, 1)	0,${R}_{pg}$− ${F}_{q}$, ${F}_{q}$+${R}_{qg}$,${C}_{ph}$− ${B}_{pr}$− ${C}_{pl}$− ${C}_{ps}$− ${F}_{s}$− ${R}_{pg}$	(0, ×, +, ×)	Unstable points	–
(0, 1, 0, 1)	0, ${R}_{qg}$− ${F}_{s}$, ${R}_{qs}$− ${F}_{q}$− ${R}_{qg}$− ${C}_{qs}$,${C}_{pl}$− ${C}_{ph}$+${C}_{ps}$+${F}_{s}$+${R}_{p}$+${R}_{pg}$	(0, ×, +, ×)	Unstable points	–
(0, 0, 1, 1)	1/(${B}_{pr}$− ${C}_{ph}$+${C}_{pl}$+${C}_{ps}$+${F}_{s}$+${M}_{p}$+ ${R}_{pg}$), 1/(${C}_{qs}$+${F}_{q}$+${M}_{q}$+${R}_{qg}$− ${R}_{qs}$), − 1/(${M}_{p}$− ${C}_{b}$+${M}_{q}$+${R}_{fg}$), − 1/(${F}_{q}$+${F}_{s}$− ${R}_{fg}$+${T}_{g}$)	(−, −, −, −)	ESS	⑤⑥ ⑦⑧
(1, 1, 1, 0)	0, 0, − ${R}_{pg}$− ${R}_{qg}$, ${C}_{ph}$− ${C}_{pl}$− ${C}_{ps}$− ${R}_{p}$	(0, 0, −, ×)	Uncertainty	–
(1, 1, 0, 1)	0, ${R}_{pg}$+${R}_{qg}$, − ${F}_{q}$− ${R}_{qg}$,${C}_{ph}$− ${C}_{pl}$− ${C}_{ps}$− ${F}_{s}$− ${R}_{p}$− ${R}_{pg}$	(0, +, −, ×)	Unstable points	–
(1, 0, 1, 1)	0, ${R}_{pg}$ − ${F}_{q}$, ${F}_{q}$+${R}_{qg}$, ${C}_{ph}$− Bpr − ${C}_{pl}$− ${C}_{ps}$− ${F}_{s}$− ${M}_{p}$− ${R}_{pg}$	(0, ×, +, ×)	Unstable points	–
(0, 1, 1, 1)	0, ${R}_{qg}$− ${F}_{s}$, ${R}_{qs}$− ${F}_{q}$− ${M}_{q}$− ${R}_{qg}$− ${C}_{qs}$,${C}_{pl}$− ${C}_{ph}$+${C}_{ps}$+${F}_{s}$+ Rp + ${R}_{pg}$	(0, ×, ×, ×)	Uncertainty	–
(1, 1, 1, 1)	0, ${R}_{qg}$− ${F}_{s}, {R}_{qs}$− ${F}_{q}$− ${M}_{q}$− ${R}_{qg}$− ${C}_{qs}$,${C}_{pl}$− ${C}_{ph}$+${C}_{ps}$+${F}_{s}$+${R}_{pg}$+${R}_{pg}$	(0, ×, +, ×)	Unstable points	–

Conditions: ①$${B}_{pr}-{C}_{ph}+{C}_{pl}+{C}_{ps}+{M}_{p}<0$$; ②$${C}_{qs}+{M}_{q}-{R}_{qs}<0$$; ③$${M}_{p}-{{\text{C}}}_{b}+{M}_{q}>0$$; ④$${F}_{q}+{F}_{s}-{R}_{fg}+{T}_{g}<0$$; ⑤$${B}_{pr}-{C}_{ph}+{C}_{pl}+{C}_{ps}+{M}_{p}+{F}_{s}+{R}_{pg}<0$$; ⑥$${C}_{qs}+{F}_{q}{+M}_{q}+{R}_{qg}-{R}_{qs}<0$$; ⑦$${M}_{p}-{{\text{C}}}_{b}+{M}_{q}+{R}_{fg}>0$$; ⑧$${F}_{q}+{F}_{s}-{R}_{fg}+{T}_{g}>0$$

### Simulation analysis

Based on the previous analysis, two points have the potential to become stable points. To further verify the validity of the evolutionarily stable points analyzed previously, the evolutionary trajectories of all gaming subjects will be numerically simulated using MATLAB 2023b in the following sections.

Seed, being the “chip” of agriculture, plays a pivotal role in ensuring the smooth operation of the agricultural system. The quality assurance of seeds is therefore of paramount importance. This section sets up simulation parameters based on real-world data to make the simulation results more reliable and reflective of the actual market dynamics. The data sources include the annual report data of Zhongnongfa Seed Group Co., Ltd. (2022)^29^, the amount of fines for counterfeit seed incidents^30^, and the income per mu of wheat planting by farmers^31^.

Seed companies are engaged in the research, development, and production of various types of crop seeds. The cost of producing high-quality seeds is denoted by ${C}_{ph}=5$. However, for higher profits, some might resort to producing counterfeit seeds at a much lower cost of ${C}_{pl}=0.01$, with additional costs such as rent-seeking from certification agencies ${B}_{pr}=0.5$, false marketing and management costs ${C}_{ps}=0.2$, and compensation to farmers for their active rights protection ${M}_{p}=0.5$, while the revenue from seed sales remains at ${R}_{p}=6$. Certification agencies, on the other hand, have a revenue of ${R}_{q}=3$ with a certification cost of ${C}_{q}=1$. They may also engage in rent-seeking activities with a revenue of ${R}_{qs}=0.5$ and a speculative cost of ${C}_{qs}=0.1$. If farmers choose to protect their rights actively, the certification agencies may have to compensate ${M}_{q}=0.1$. Farmers incur a seed purchase cost of ${C}_{f}=6$ and can earn a revenue of ${R}_{f}=7.8$ post a successful harvest. However, in case they end up buying counterfeit seeds, they suffer a loss of ${H}_{f}=6.5$ with an active rights protection cost of ${C}_{b}=1$. The governmental departments bear a cost of ${C}_{g}=4$ for regulation. Under strict regulation, when high-quality seeds enter the market, seed companies and certification agencies are rewarded with ${R}_{pg}=0.2$ and ${R}_{qg}=0.1$ respectively, while the societal benefit is ${R}_{g}=7$. If counterfeit seeds enter the market, fines of ${F}_{s}=1$ and ${F}_{q}=0.1$ are imposed on seed companies and certification agencies respectively, with a reward of ${R}_{fg}=1.5$ for farmers' active rights protection. The cost of market rectification and maintaining social stability by the government is denoted by ${D}_{g}=10$. The baseline parameter values are set to satisfy conditions ⑤ to ⑧. The initial strategy choices of all game subjects are set at $x=0.5$, $y=0.5$, $z=0.5$, $u=0.5$.

#### Influence of cost–benefit ratio on seed companies

**Figure 3:**
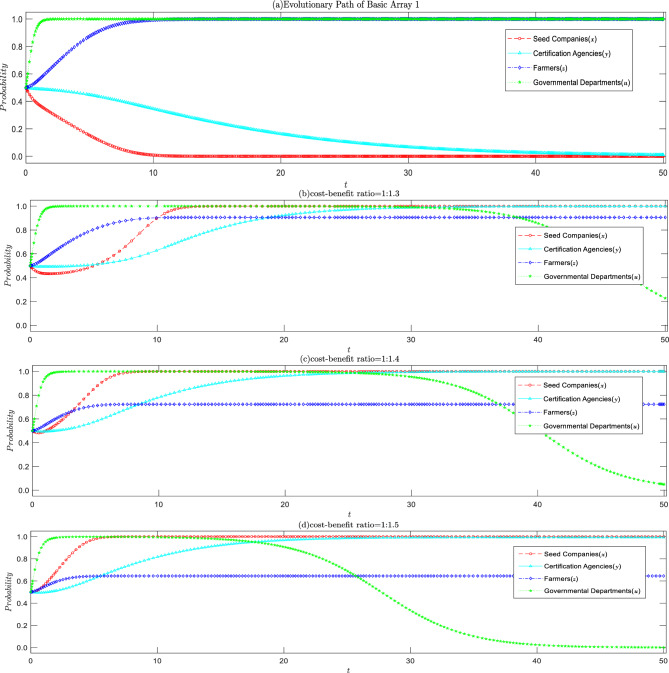
Impact of changes in cost–benefit ratio of seed companies on the evolutionary strategy of all parties.

When conditions ⑤ to ⑧ are satisfied, the basic array 1 sets the cost–benefit ratio to 1:1.2, based on the annual report data released by the Agricultural Development Seed Group Co., Ltd. The evolution game process and the outcomes are illustrated in Fig. 3a, where the system gradually stabilizes at (0, 0, 1, 1), representing the stable strategy of “seed companies producing inferior seeds, certification agencies inclined to rent-seeking, farmers actively asserting their rights, and strict government regulation”. This validates the accuracy of the conclusions drawn earlier and provides a more intuitive explanation of the issue at hand. As depicted in Fig. 3b–d, an increase in the cost–benefit ratio accelerates the speed at which seed companies lean towards producing high-quality seeds, and the governmental departments are more inclined to opt for lax regulation, thus achieving the maximum social benefit at the lowest management cost. This is in alignment with the conclusions drawn in Inference 1.

#### Influence of rent-seeking costs on certification agencies

**Figure 4:**
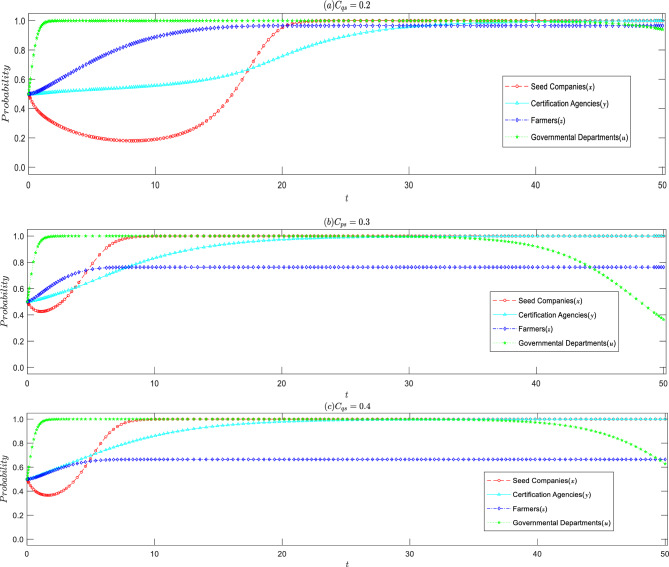
Impact of increased rent-seeking cost of certification agencies on the evolutionary strategy of all parties.

Compared with the basic array 1, it can be observed that the higher the speculative cost, the greater the probability of the certification agencies refusing rent-seeking. This is consistent with the conclusion of Corollary 2. Moreover, as the speculative cost increases, the certification agencies tend to refuse rent-seeking more quickly, and seed companies tend to produce high-quality seeds faster, as shown in Fig. 4. When seed companies produce inferior seeds, appraisal agencies refuse to seek rent and prevent inferior seeds from entering the market, resulting in silence costs for seed companies. Therefore, as the probability of the certification agencies refusing rent-seeking increases, seed companies lower the probability of producing inferior seeds.

#### Influence of farmers' rights protection cost

**Figure 5:**
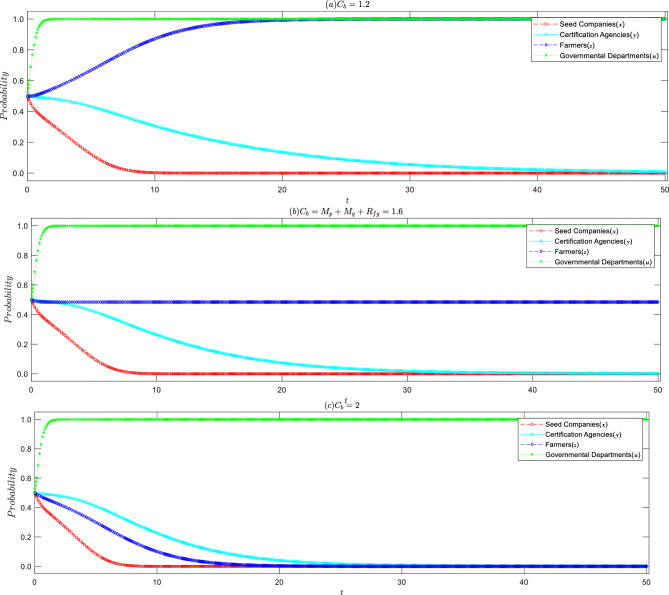
Impact of farmers' litigation costs on evolutionary strategies of all parties.

When seed companies produce inferior seeds and the certification agencies have an intention to seek rent, the interests of farmers who purchase inferior seeds are harmed. At this time, whether the farmers take the initiative to protect their rights is affected by the cost of rights protection, as shown in Fig. 5. When the cost of rights protection is less than the sum of compensation and rewards, the probability of farmers taking the initiative to protect their rights continuously increases over time. When the cost of rights protection is equal to the sum of compensation and government rewards, i.e., ${C}_{b}={M}_{p}+{M}_{q}+{R}_{fg}$, the probability of farmers actively protecting or giving up their rights is 50% each, being in a non-stable evolutionary equilibrium, where any minor change will cause it to deviate towards the happening state. The higher the cost of rights protection, even if rights protection can recover losses, the probability of farmers giving up rights protection increases with the evolution of time.

#### Impact of government regulatory mechanism

**Figure 6:**
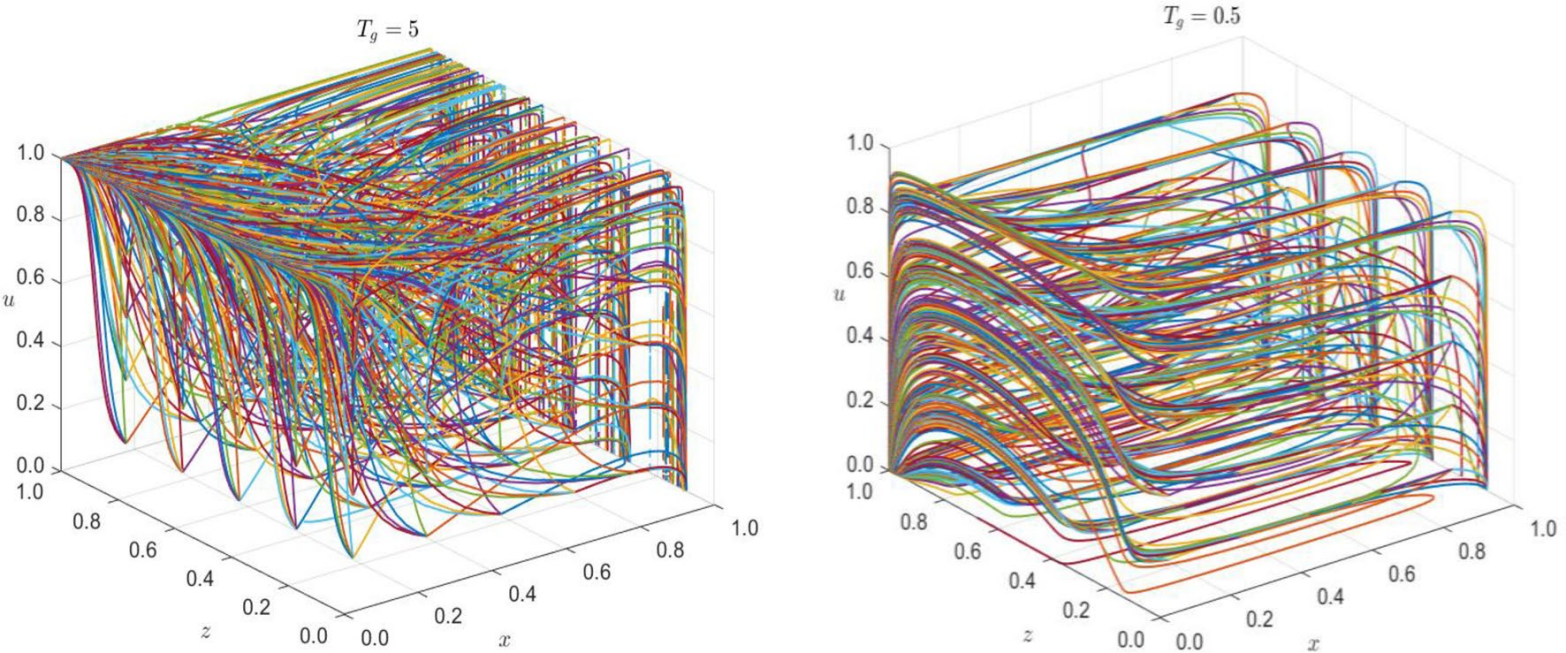
Impact of higher-level accountability intensity on the evolutionary strategies of governmental departments.

To further validate the feasibility and effectiveness of the governmental departments on seed quality assurance, values in Array 1 are altered, setting $Fs=0.1$ and $Tg=0.5$, to satisfy conditions ① to ④, forming Array 2. Based on Array 1 and Array 2, a simulation is conducted to analyze the impact of the accountability strength of the higher-level government on the strategy choice of the governmental departments, as illustrated in Fig. 6. This simulation serves both as an analysis of the evolutionary equilibrium point and an affirmation of the function of the higher-level accountability mechanism.

## Discussion

### Evolutionary game equilibrium point analysis

This study highlights the crucial roles of seed companies, certification agencies, farmers, and government in ensuring seed quality. Under the influence of varying economic motives and degrees of regulation, the production of high-quality seeds and the rejection of rent-seeking are crucial to seed quality assurance. In essence, the combined influence of market forces and regulatory measures forms the cornerstone of seed quality assurance. (1) The equilibrium points, (0, 0, 1, 1) and (0, 0, 1, 0), in this paper all occur under the environment where the seed companies produce inferior seeds and the certification agencies intend to seek rent, causing the inferior seeds to enter the market. In this scenario, farmers tend to choose strategies that protect their own interests, while the government makes different choices under different circumstances. (2) According to the cost–benefit analysis, if seed companies produce high-quality seeds, regardless of whether the certification agencies intend to seek rent, whether the farmers actively uphold their rights, or whether the governmental departments are strictly regulated, high-quality seeds will bring sufficient benefits to farmers, the government, and society^32^. (3) If seed companies produce inferior seeds, and the certification agencies refuse to seek rent, thus preventing the inferior seeds from entering the market, the interests of the farmers are not harmed. Therefore, the strategy choices of the farmers or the government have no effect on quality assurance^33^. (4) If inferior seeds enter the market, according to the equilibrium point analysis, the interests of the farmers are directly impacted. Upholding their rights is the best strategy choice for the farmers. The increase in social instability factors leads to a scenario where the government's strategy choice depends on the comparison between the sum of the rewards for farmers’ rights protection and the income from superior accountability and fines.

### Stakeholder strategy choice impact mechanisms

The strategic choices of seed companies are influenced by a multitude of factors including speculative costs, production costs, and governmental policies. Profit maximisation, serving as a significant economic motive, directly influences production decisions; a higher cost–benefit ratio inclines seed companies towards the production of high-quality seeds. Production cost is also a significant influencing factor; greater production costs may prompt seed companies to opt for the production of lower-cost substandard seeds. Governmental rewards and penalties also affect company decisions^34^. The encouragement for the production of high-quality seeds can be fostered by amplifying rewards, whilst the deterrence of seed companies from unlawful activities can be achieved by intensifying penalty measures.

The rent-seeking behaviour of certification agencies is conjointly influenced by rent-seeking costs, anticipated illegal compensation, and rent-seeking benefits. Rent-seeking costs directly affect the rent-seeking actions of certification agencies; higher costs erode higher profits, thus reducing the economic benefits and leading to a decline in the willingness to engage in rent-seeking. Anticipated illegal compensation is also a critical factor; higher expected compensation augments the risks and costs associated with rent-seeking behaviour, thereby increasing the probability of certification agencies refusing rent-seeking. Rent-seeking benefits, serving as the driving force behind the rent-seeking actions of certification agencies, also impact their decisions^35^. Higher speculative benefits, despite certain risks and costs, diminish the probability of certification agencies refusing rent-seeking.

The degree of refinement in the compensation mechanism directly impacts the proactiveness of farmers in seeking legal redress^36,37^. Should farmers be able to obtain reasonable and ample compensation from seed companies and certification agencies, they would be more inclined to resolve issues through legal channels. When the rights of farmers are infringed upon, higher litigation costs may deter them from taking legal action. Government support for farmers' rights protection, such as legal aid, informational services, and simplified litigation procedures, also significantly influence the farmers' decision on whether to pursue legal action^38^.

The strategy choices of government regulatory departments are collectively influenced by fine mechanisms, accountability mechanisms, and reward mechanisms. The fine mechanism is a crucial component of the regulatory strategy, where moderate fines can prevent and mitigate illegal activities by seed companies and certification agencies, thereby maintaining market fairness and stability. The higher-level accountability mechanism is another non-negligible factor; strengthening this mechanism can enhance the execution efficiency of regulatory departments, ensuring the effective implementation of regulatory policies^38^. The reward mechanism also plays a pivotal role, encouraging seed companies to allocate more resources towards the production of high-quality seeds, thereby promoting the high-quality development of seeds.

### Limitations and future research directions

Intellectual Property Rights (IPRs) play a pivotal role in safeguarding the rights of seed patent holders throughout the processes of seed research, development, production, and sales. In this study, the production of inferior seeds by seed companies significantly infringes upon the rights of patent holders, potentially leading to complaints from other seed enterprises. Future research needs to incorporate the impact of this dimension. Seed companies' strategic shifts solely from a cost–benefit perspective, variations in government incentives for producing high-quality seeds, fines for producing inferior seeds, rent-seeking costs, speculative costs, and compensations for farmer litigations will all exert influences on the strategic choices of the gaming stakeholders. Compared to the factors analyzed in this paper, these elements exert a lesser impact on strategy selection, yet they should be integrated into subsequent studies on seed companies.

Given that seed companies are the starting point of the seed quality assurance system, and that much of the research inadequacies lie in the production phase of high-quality seeds, only external factors affecting seed companies have been considered, overlooking the impact of endogenous dynamics. Therefore, future research will unfold from the internal driving mechanisms of seed companies, combining complex network and dynamical studies to construct an evolutionary game model for the complex network of seed companies. This will entail analyzing the influence of imitation effects and demonstration effects on seed companies’ inclination towards the production of high-quality seeds.

## Conclusion

This study unveils the intricate game relationships among key stakeholders in the seed industry within agricultural systems, and the mechanism of influence regarding strategic choices. The assurance of seed quality is delineated into three stages: firstly, the production stage, where the genesis of seed quality assurance lies in the choice of seed companies to produce high-quality seeds. Under the joint influence of farmer feedback and regulatory bodies, the emergence of high-quality seeds fundamentally safeguards the development of seed quality at its source. Secondly, the market entry stage, where substandard seeds, once produced, are prevented from entering the market. In this context, regulatory measures come into play, and certification agencies act as a “barrier” to market entry for these inferior seeds. Thirdly, the feedback stage, where substandard seeds, if they enter the market, market forces come into play and will face the critical scrutiny of farmers who are essential to seed quality assurance. Seed companies are the starting point of the seed quality assurance system, and ensuring quality at the “source” can eliminate and reduce regulatory costs in subsequent stages. Certification agencies play a pivotal role in seed quality assessment, with their fairness and accuracy directly impacting the healthy development of seeds. Farmers are the feedback providers against substandard seeds and beneficiaries of high-quality seeds. The government acts as a “guardian” in quality assurance, ensuring a conducive environment for quality control. Through the combined effects of market forces and regulatory frameworks, and with concerted efforts from all stakeholders involved, an effective seed quality assurance system can be refined, thereby ensuring the sustainable development of agricultural systems.

## Data Availability

The data that support the findings of this study are available from the corresponding author upon reasonable request.
